# Comparison of methods of isolating extracellular vesicle microRNA from HepG2 cells for High-throughput sequencing

**DOI:** 10.3389/fmolb.2022.976528

**Published:** 2022-09-26

**Authors:** Ying-Hui Xiong, Xue-Gong Fan, Ya-Yu Chen, Yan Huang, Jun Quan, Pan-Pan Yi, Mei-Fang Xiao, Ze-Bing Huang, Xing-Wang Hu

**Affiliations:** ^1^ Department of Infectious Diseases, Xiangya Hospital, Central South University, Changsha, China; ^2^ Hunan Key Laboratory of Viral Hepatitis, Xiangya Hospital, Central South University, Changsha, China; ^3^ Department of Health Management Center, Xiangya Hospital, Central South University, Changsha, Hunan, China; ^4^ National Clinical Research Center for Geriatric Disorders, Xiangya Hospital, Central South University, Hunan, China

**Keywords:** extracellular vesicles isolation, microRNA, High-throughput sequencing, HepG2 cells, cell culture medium

## Abstract

**Background:** Extracellular vesicles (EVs) were reported to participate in various cellular processes based on the biomolecules, particularly microRNAs. Numerous commercial EVs isolation reagents are available. However, whether these reagents are suitable for separating EVs from the culture medium supernatant supernatant of model cell lines, such as HepG2, and whether the isolated products are suitable for High-throughput sequencing remains unclear.

**Methods:** We examined three commonly used EVs isolation kits: the ExoQuick-TC exosome precipitation solution (EQ), Total Exosome Isolation from cell culture medium (EI), and exoEasy Maxi Kit (EM), to isolate EVs from HepG2 cell culture medium supernatants. EVs were identified based on marker proteins, particle size measurements, and electron microscopy analysis. The total amounts of microRNA and microRNA High-throughput sequencing data quality from EVs isolated by each kit were compared.

**Results:** The total amount of EVs’ microRNA isolated from the EI and EM groups were higher than that obtained from the EQ group (EQ/EI: *p* = 0.036, EI/EM: *p* = 0.024). High-throughput sequencing data quality evaluation showed that the EI group possessed higher quality than those in the EM group.

**Conclusion:** For the cell culture medium from HepG2, EVs’ microRNA isolated by EI reagents might be more suitable for High-throughput sequencing applications.

## Introduction

Extracellular vesicles (EVs) are membranous vesicles with a lipid bilayer released by all organisms and all cells into various body fluids under physiological and pathological conditions (Milane et al., 2015; Zhao et al., 2017; Thery et al., 2018). During EVs formation, various cellular molecules, such as nucleic acids, peptides, proteins, and lipids are loaded into EVs via passive or active pathways (Valadi et al., 2007; Melo et al., 2014). Over 4,600 proteins, including cytosolic, membrane, Golgi apparatus, and endoplasmic reticulum proteins have been isolated and identified from EVs (Mathivanan et al., 2010; Murdica et al., 2019). EVs can also carry large amounts of nucleic acids (Qing et al., 2018; Cheng et al., 2019; Luo et al., 2019), including microRNA (miRNA), long noncoding (lncRNA) and so on. Increasing evidence has demonstrated that EVs can mediate horizontal information exchange (Fruhbeis et al., 2013; Li et al., 2013), changes in the cellular microenvironment, and long-distance tissue-cell interactions by delivering their encapsulated molecules to recipient cells (Costa-Silva et al., 2015), affecting processes such as angiogenesis, immune regulation, and cancer cell growth and metastasis (Wei et al., 2019; You et al., 2019).

MiRNA is a type of small noncoding RNA (19–25 nucleic acids long) present in both cells (Ambros, 2004)and EVs. Recent studies have shown that specific plasma EVs’ miRNAs (such as EVs miR-122) in the patients with various liver diseases are involved in physiological and pathological processes in liver cells (Jiao et al., 2017; Mosedale et al., 2018). Previously, we found that the supernatant of culture medium used to grow HepG2 expressing hepatitis B virus X protein (HBx) promoted the proliferation of non-HBx-expressing HepG2 cells. Considering the vital roles of EVs in cellular communication, HBx may promote the proliferation and metastasis of liver cancer cells by regulating the EVs pathway. However, the best way to efficiently and rapidly isolate high-quality EVs’ miRNAs from HepG2 cell culture medium supernatants for High-throughput sequencing analysis remains unclear.

Currently, EVs isolation methods include ultracentrifugation, immunomagnetic beads, chromatography, ultrafiltration centrifugation, and poly (ethylene glycol)-based precipitation (Clayton et al., 2001; Thery et al., 2006; Delic et al., 2016; Hu et al., 2016). Above all can be performed to separate EVs but with limitations, including requiring specialized equipment, large sample sizes, and advanced operational and technical skills. In recent years, various commercial EVs isolation reagents have been introduced, allowing rapid and convenient EVs isolation from various samples. These kits do not require special equipment and show high isolation efficiency and purity, and thus have become favored by researchers. However, whether these kits were suitable for EVs extraction form HepG2 cell culture medium supernatants or the EVs’ miRNA isolated by these kits is suitable for High-throughput sequencing remains unclear. Thus, this study was conducted to compare three different commercial EVs isolation reagents commonly used by researchers, which separate the EVs based on different principles, providing guidance for the rapid isolation of high-quality EVs’ miRNA from HepG2 cell culture medium supernatants to obtain reliable sequencing results, paving the way for further research.

## Results

### Verification of EVs in HepG2 cell culture medium supernatants

**FIGURE 1:**
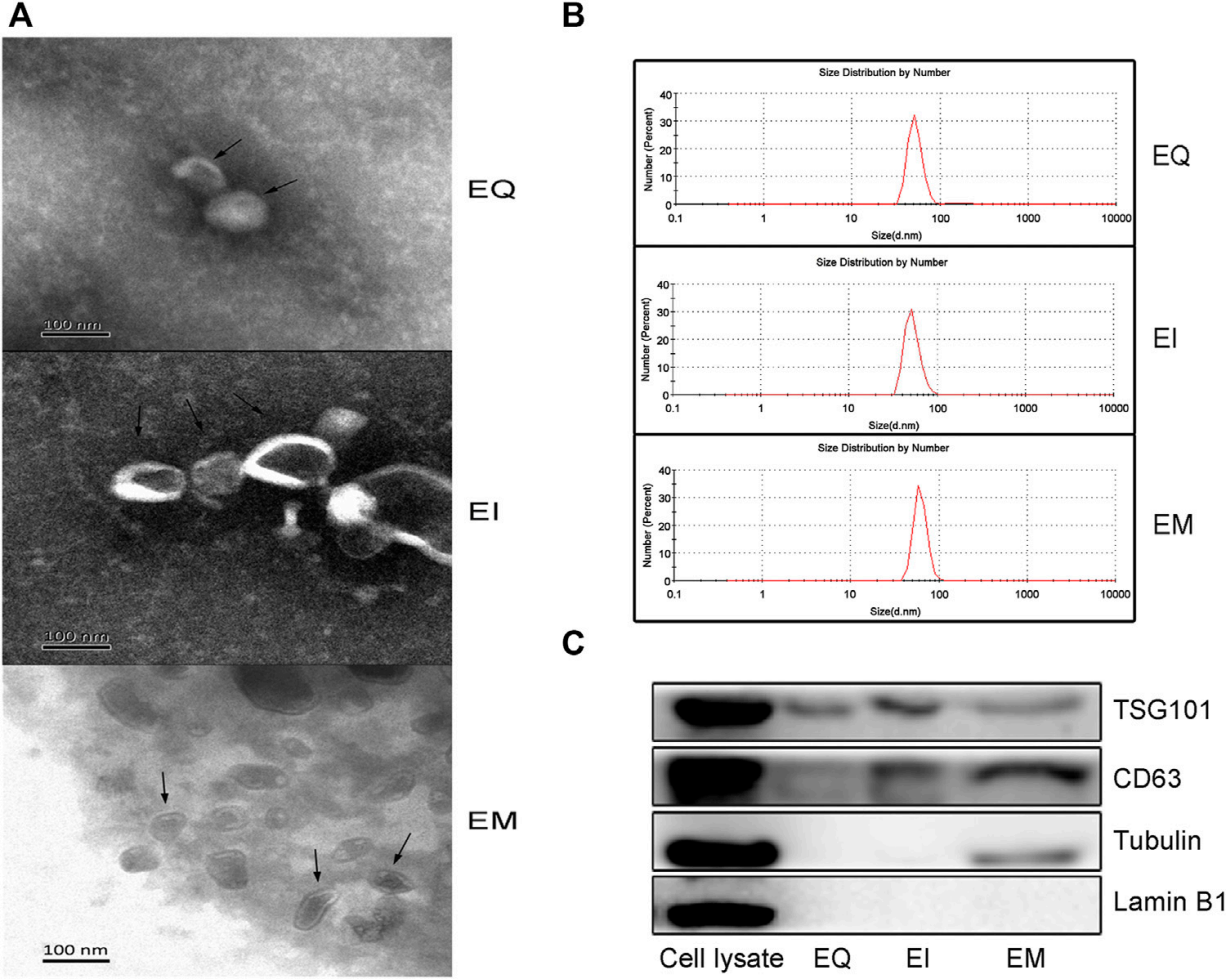
Verify EVs isolated by three EVs isolation kits, EQ, EI, and EM. 8 ml of the cell culture medium supernatant was collected from HepG2 (1.01×10^7^ cells per dish), and 13.3 ml supernatant was individually applied for EVs isolation by each kit. Triple samples were analyzed for each group. **(A)** Transmission electron microscopy images of isolated HepG2 cell-derived EVs (arrows). **(B)** Nanoparticle size analyzer measurements of the size distribution ranges of isolated HepG2 cell-derived EVs. The diameter distribution of EQ, EI and EM group is respectively 51.4–94.9 nm, 52.5–100 nm and 60.1–105.1 nm. **(C)** Western blot analysis of TSG101 and CD63 expression levels in isolated HepG2 cell-derived EVs.EVs purity was measured by analyzing the expression levels of Tubulin and Lamin B1. 40 μl protein from EVs separated by each kit was loaded.

According to the guidelines of and MISEV2018 (Thery et al., 2018), Transmission electron microscopy, particle size measurements, and western blotting for the EVs marker proteins (TSG101, CD63) and proteins for assessing the degree of purity of the EVs preparation (Tubulin, Lamin B1) were performed. As shown in Figure 1A, the EVs isolated by the three reagents were spherical, hemispherical, or cup-shaped, with an intact vesicular structure. The particle diameter distributions of the three groups were 40–100 nm (Figure 1B). As shown in Figure 1C, western blot analysis revealed bands specific to the molecular weights of TSG101 and CD63 in the EQ, EI, and EM groups, no Lamin B1 bands in any group, and Tubulin bands in the EM group but not in the EQ and EI groups.

### Comparison of the amounts of EVs’ miRNAs extracted by different reagent kits

**FIGURE 2:**
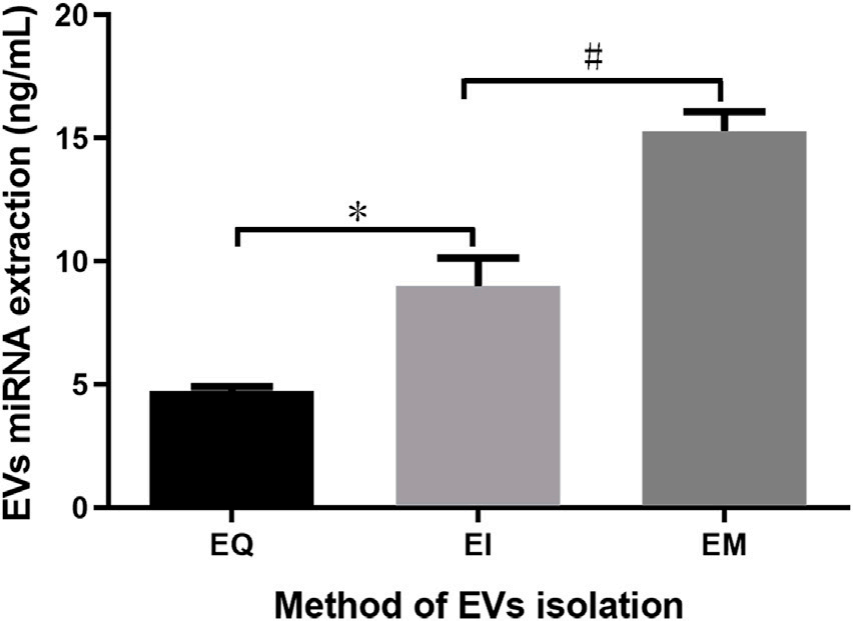
Comparison of total EVs’ miRNAs from the EQ, EI, and EM groups. 8 ml of the cell culture medium supernatant was collected from HepG2 (1.01×10^7^ cells per dish), and 13.3 ml supernatant was individually applied for EVs’ miRNAs isolation by each kit. Duplicate samples were analyzed for each group. The total amounts of EVs’ miRNAs per 1 ml supernatant were measured by ultraviolet spectrophotometry. *, *p* < 0.05; #, *p* < 0.05.

After using the miRNeasy Serum/Plasma Kit to extract EVs’ miRNAs from EQ, EI, and EM groups, the miRNA was quantified. The amounts of EVs’ miRNAs per 1 ml cell culture medium supernatant were 4.74 ± 0.17, 8.99 ± 1.16, and 15.28 ± 0.79 ng for the EQ, EI, and EM groups, respectively. The amounts of EVs’ miRNAs in the EI and EM groups were significantly higher than in the EQ group (QI/EI: *p* = 0.036, EI/EM: *p* = 0.024, Figure 2).

### Comparison of High-throughput sequencing data quality of EVs’ miRNAs extracted with different kits

**FIGURE 3:**
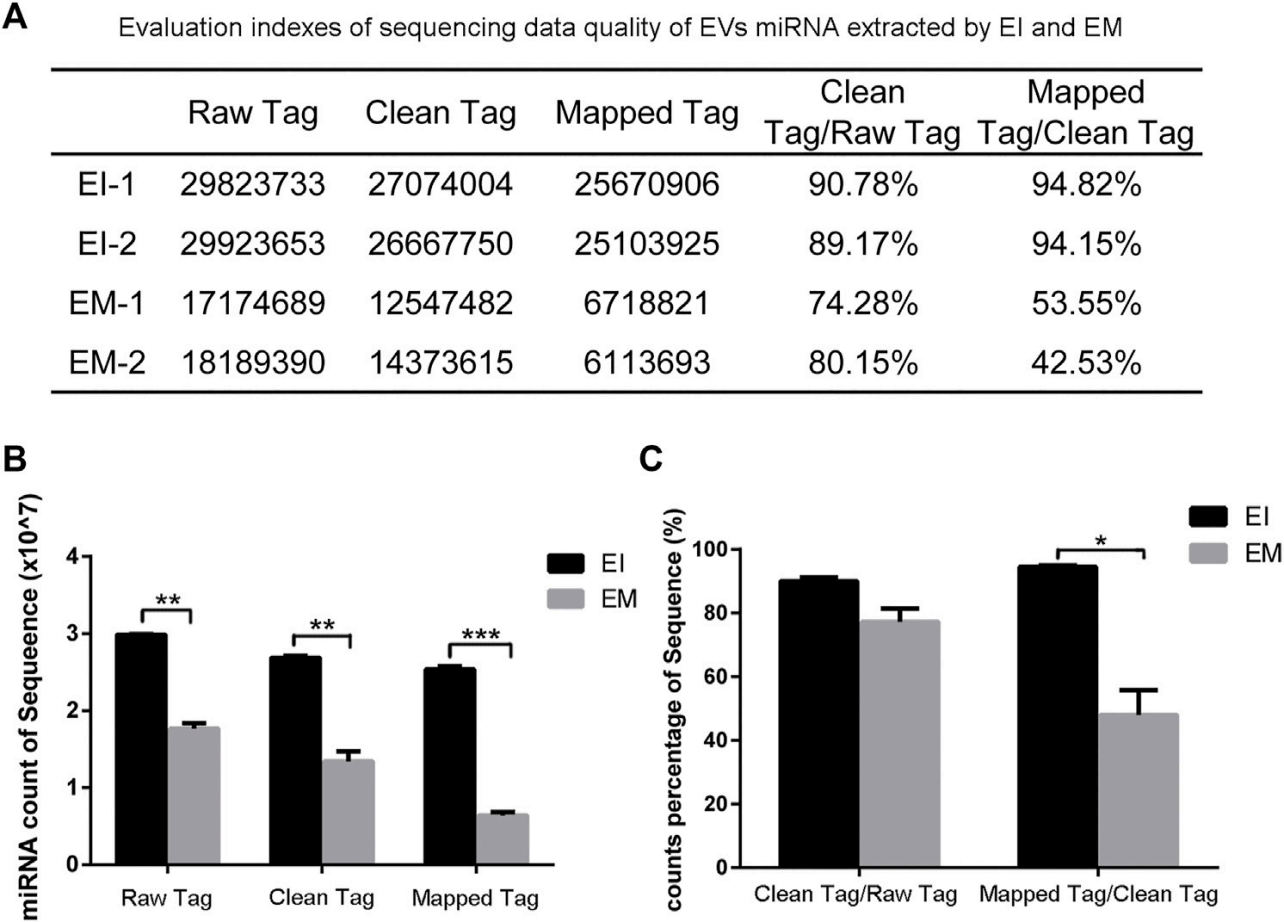
Analysis of EVvs’ miRNAs sequencing data from the EI and EM groups. 8 ml of the cell culture medium supernatant was collected from HepG2 (1.01×10^7^ cells per dish), and 13.3 ml supernatant was individually applied for EVs’ miRNAs isolation by each kit. Duplicated samples were analyzed for each group. High-throughput sequencing was performed for EVs’ miRNAs from the EI and EM group. **(A)** Statistical analysis results of two sets of sequencing data. **(B,C)** High-throughput sequencing data quality comparison between the two groups. *, *p* < 0.05, **, *p* < 0.01, ***, *p* < 0.001.

**FIGURE 4:**
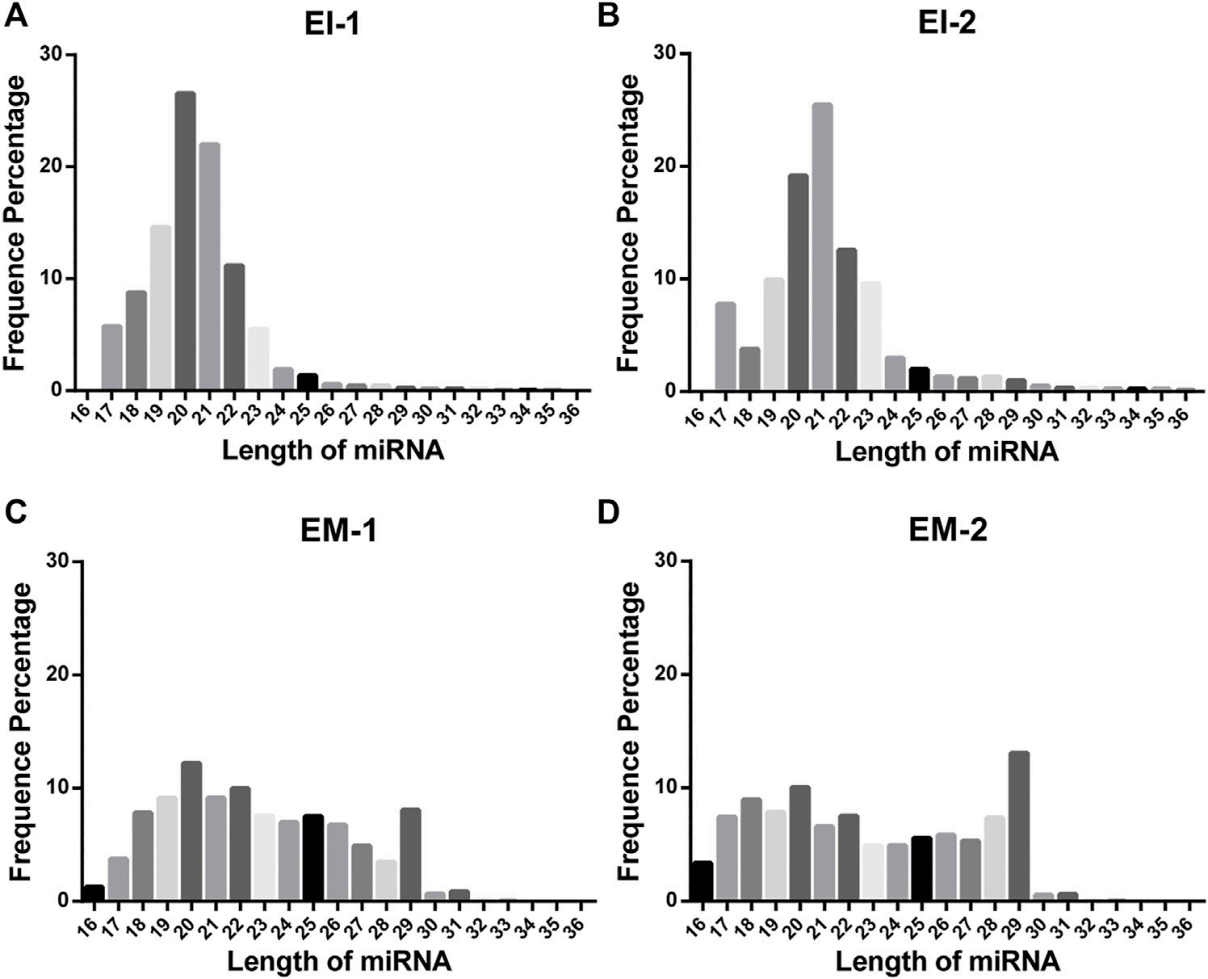
Length distribution of EVs’ miRNAs from the EI and EM groups. 8 ml of the cell culture medium supernatant was collected from HepG2 (1.01×10^7^ cells per dish), and 13.3 ml supernatant was individually applied for EVs’ miRNA isolation by each kit. Duplicate samples were analyzed for each group. High-throughput sequencing was performed for EVs’ miRNAs from the EI and EM group. **(A–D)** Nucleic acid length distributions of EVs’ miRNAs isolated by the EI and EM kit.

We constructed a miRNA library and performed the High-throughput sequencing analysis of the miRNA extracted from the EVs isolated by the EI and EM kits. As shown in Figure 3, quality of miRNA sequencing was analyzed. The EVs’ miRNAs sequencing data quality evaluation showed that the parameters of the EI group were higher than those of the EM group, indicating that the sequencing data quality of the EI group was superior to that of the EM group. [The parameters include Raw Tag (original complete reads obtained with the sequencer, *p* = 0.0017), Clean Tag (reads for which data quality met the quality control parameters, *p* = 0.0048), Mapped Tag (reads successfully mapped to the genome database, *p* = 0.0005), Mapped Tag/Clean Tag (*p* = 0.0138), and Clean Tag/Raw Tag (*p* = 0.0525)] Furthermore, as shown in Figure 4, miRNA lengths in the EI group were mainly 17–23 nt. The proportions of miRNA with a length of 19–25 nt (the range of miRNA length) were 83.03% for EI-1 and 73.05% for EI-2. MiRNA lengths in the EM group were more evenly distributed at 17–29 nt, and the proportion of miRNA with a length distribution of 19–25 nt was 47.36% for EM-1 and 62.37% for EM-2. This suggested that the length distribution pattern of EVs’ miRNAs obtained by the EI kit was more typical than the EM kit.

## Discussion

Recent studies have shown that HBx regulates EVs secretion (Kapoor et al., 2017). Therefore, determining whether HBx can promote the malignant transformation of hepatocytes by regulating changes in EVs’ miRNAs and other components is vital for comprehensive understanding of the role and pathogenesis of Hepatitis B Virus (HBV) in hepatitis and hepatitis B related liver cancer. However, EVs are released from all organs and different cell types, making a significant change in EVs induced by dysregulated genes in one specific cell type obscured by noise from other EVs. Therefore, constructing cell-line-based disease models is vital to explore the role of EVs in liver diseases. Based on our need, we chose the HepG2 as the model cell to investigate whether commonly used isolation reagents are suitable for isolating EVs’ miRNA for High-throughput sequencing.

The BGISEQ-500 sequencing system developed by BGI (Fang et al., 2018) was used to sequence EVs’ miRNAs from the cell culture medium supernatant. Because of the vast amount of information obtained from trace sources, sample quality is critical for ensuring successful sequencing. To obtain sufficient and high-quality EVs’ miRNAs from HepG2, we identified EVs, quantified the amount of EVs’ miRNA, and performed a quality assessment of the EVs miRNAs sequencing data to ensure the methods selected for EVs’ miRNAs isolation are suitable for High-throughput sequencing.

We evaluated the three commonly used commercial EVs isolation reagents which were developed based on different principles. Transmission electron microscopy and particle size measurement of the EVs showed that the products of the three kits had typical EVs characteristics, including a hemispherical or cup-like shape, bilayer vesicular structure, and diameters of approximately 40–100 nm. The specific EVs marker proteins TSG101 and CD63 were detected by western blot analysis (Wang et al., 2015). These results suggest that all three methods can successfully isolate EVs.

After EVs were obtained, we extracted EVs’ miRNAs using the miRNeasy Serum/Plasma Kit. To avoid possible interference from free RNAs (particularly miRNAs), which may affect subsequent measurements, we first isolated EVs and then extracted the EVs’ miRNAs. The phospholipid bilayer of the EVs protects miRNA from RNase degradation (Kalra et al., 2013; Ge et al., 2014). Therefore, RNase was not removed during operations before disrupting the EVs to release its miRNA, so that free miRNA naturally degrades during the process. As shown in Figure 2, quantitative analysis of the isolated EVs’ miRNAs indicated that more miRNA could be obtained using the EI and EM kits. Therefore, these two kits were used to isolate EVs for subsequent High-throughput EVs miRNA sequencing.

High-throughput sequencing result was consisted of vast sequencing reads. The 3′ ends of the reads contain a tag for the linker sequence fragment with a variable length. By comparing the tag sequence used with a known database, low-quality or incomplete reads can be removed. Therefore, the number of tags is a useful index for assessing sequencing quality. In post-processing, data that overlap are often clustered. These clusters must be mapped to genomic data to analyze their function and significance. Therefore, Tag parameters, including Raw Tag, Clean Tag, Mapped Tag, Clean Tag/Raw Tag, and Mapped Tag/Clean Tag values, are critical indicators for evaluating sequencing data quality (SEQC/MAQC-III Consortium, 2014). And these parameters were higher for the EI group than the EM group. The pattern of miRNA length distribution suggests that the small RNAs length distribution of the EI group was more concentrated in the range of miRNA length range (19–25 nt) than those of the EM group.

Taken the aspects of miRNA amount, sequencing data quality and miRNA length distribution together, the EI kit might be more suitable to extract EVs’ miRNAs for High-throughput sequencing application. Probably, our findings could provide some hints for researchers in selecting preferable EVs extraction methods to obtaining reliable High-throughput sequencing results from cell culture medium derived EVs’ miRNAs.

However, there were some limitations to our study. Firstly, only three kits, as well as, only EVs derived from HepG2 cell culture medium was tested. It remains unclear whether these results apply to other kits and cell lines. Secondly, we only compared EVs’ miRNAs, but not other nucleic acid molecules in EVs, such as lncRNAs and circular RNAs. Thirdly, we analyzed the EVs’ miRNAs using only one sequencing platform. Whether the products extracted by this method are equivalent for other sequencing platforms requires further analysis.

## Materials and methods

### Cell lines, cell culture medium, antibodies, and other reagents

The HepG2 cell line was purchased from the *ATCC.* Dulbecco’s modified Eagle’s medium (DMEM; Gibco, Grand Island, NY, United States) containing 10% fetal bovine serum (Gibco) was used to culture HepG2 cells. The following EVs isolation kits were used: ExoQuick-TC exosome precipitation solution (EXOTC50A-1, System Biosciences, Palo Alto, CA, United States), Total Exosome Isolation (from cell culture medium; 4478359, Invitrogen, Carlsbad, CA, United States), and exoEasy Maxi Kit (76064, EM, Qiagen, Hilden, Germany). After EVs isolation, EVs proteins were extracted with RIPA lysis buffer (Sangon Biotech, Shanghai, China) supplemented with protease inhibitor cocktail (Selleck Chemicals, Houston, TX, United States) for western blot analysis using rabbit polyclonal anti-CD63 (A5271, ABclonal Technology, Woburn, MA, United States) and rabbit anti-tumor susceptibility 101 (TSG101) (HPA006161, Sigma-Aldrich, St. Louis, MO, United States) antibodies.

### Cell culture and harvest of culture medium supernatant

HepG2 cells were seeded into cell culture dishes (10 cm of diameter) and washed with sterile 1× phosphate-buffered saline (PBS), and then serum-free DMEM was added. After 48 h, 8 ml of the cell culture medium supernatant was collected from HepG2 (1.01×10^7^ cells per dish) by centrifuging at 3,000 g for 15 min and then filtered to exclude particles larger than 0.8 μm with Millipore Millex-AA syringe filters (SLAA033SB, Merck KGaA, Darmstadt, Germany). The supernatant was kept at 4°C for subsequent EVs isolation or at −80°C for long-term storage.

### Extraction of EVs from cell culture medium supernatants with the EQ, EI, and EM reagent kits

Respectively, the equal volume of HepG2 cell culture medium supernatant (13.3 ml) was used for EVs isolation by the ExoQuick-TC exosome precipitation solution (EQ), Total Exosome Isolation (EI), and exoEasy Maxi Kit (EM) exosome isolation reagents. The kits’ instructions were briefly clarified as follows:

EQ: 1) Add ExoQuick-TC Exosome Precipitation Solution to the culture medium supernatant at the volume ratio of 1:5. Mix well by gently inverting the tube 5 times. Then, keep the mixture at 4°C for 16 h.
2) Centrifuge the mixture at 1,500 g for 30 min at 4°C and discard the supernatant. Re-centrifugate at 1,500 *g* for 5 min at 4°C to remove residual ExoQuick-TC solution, taking great care not to disturb the precipitated EVs in pellet.
3) Resuspend EVs pellet in 100 μL 1× PBS and keep it at 4°C until next procedure.
EI: 1) Add Total Exosome Isolation reagent to the culture medium supernatant at the volume ratio of 1:2. Mix well by gently inverting the tube 5 times. Then, keep the mixture at 4°C for 16 h.
2) Centrifuge the mixture at 10000 g for 60 min at 4°C and discard the supernatant. Re-centrifugate at 1,500 *g* for 5 min at 4°C to remove residual reagent, taking great care not to disturb the precipitated EVs in pellet.
3) Resuspend EVs pellet in 100 μL 1× PBS and keep it at 4°C until next procedure.
EM: 1) Add 13.3 ml buffer XBP to the culture medium supernatant. Mix well by gently inverting the tube 5 times. Then keep the mixture at room temperature for 30 min.
2) Add the mixture onto the exoEasy spin column and centrifuge at 500 g for 1 min. Discard the flow-through, and place the column back into the same collection tube and then centrifuge at 5,000 g for 1 min to remove residual liquid from the membrane.
3) Add 10 ml buffer XWP and centrifuge at 5,000 g for 5 min.
4) Transfer the spin column to a fresh collection tube. Add 1 ml Buffer XE to the membrane and incubate for 1 min. Centrifuge at 500 g for 5 min to collect the eluate.
5) Re-apply the eluate to the exoEasy spin column membrane and incubate for 1 min. Centrifuge at 5,000 g for 5 min to collect the eluate.
6) The eluate was concentrated through a 50-kD Amicon^®^ Ultra-0.5 Centrifugal Filter Devices (UFC505096, Merck KGaA, Darmstadt, Germany) with 7,500 g for 10 min. Add 1×PBS into the concentration to yield total 100 μl EVs suspension, then store at 4°C until use.

### Western blot detection of EVs marker proteins

Briefly, EVs suspension was mixed with 2× RIPA buffer at a 1:1 ratio and placed on a shaker for 30 min (4°C). The mixtures were centrifuged at 20,000 g for 20 min (4°C) to obtain lysed EVs proteins. TSG101, CD63, Lamin B1 and Tubulin were detected by western blot analysis after separating the proteins (40 μl) by 10% SDS-PAGE (sodium dodecyl sulfate-polyacrylamide gel electrophoresis).

### EVs particle size measurements

The 10 μl EV suspension was loaded to a nanoparticle size and potential analyzer (Malvern Instruments, Malvern, United Kingdom) and measured according to the manufacturer’s instructions.

### Transmission electron microscopy of EVs

The EVs suspensions were sent to the Department of Electron Microscopy, Xiangya Hospital, Central South University, for adsorption and fixation on a formvar-carbon mesh copper grid, followed by transmission electron microscopy analysis.

### EVs’ miRNAs extraction and concentration determination

On the aseptic/nuclease-free bench, EVs’ miRNAs were isolated using the miRNeasy Serum/Plasma Kit (217184, Qiagen) according to the manufacturer’s instructions. The miRNA concentration was determined with a Nano-200 Ultrafine nucleic acid analyzer (ALLSHENG, Hangzhou, China). The extracted miRNA was stored at −80°C.

### EVs’ miRNAs library construction and High-throughput sequencing

High-throughput sequencing of EVs’ miRNAs was performed by BGI Genomics (Shenzhen, China) on a BGISEQ-500 sequencing platform.

### Statistical analysis

All data were analyzed with GraphPad Prism version 8.0.0 software (GraphPad Software, San Diego, California, United States). The *t*-test was used. *p* < 0.05 was considered statistically significant.

## Data Availability

The datasets presented in this study can be found in online repositories. The names of the repository/repositories and accession number(s) can be found below: https://www.ncbi.nlm.nih.gov/, PRJNA879871.
